# Neurogenesis and Specification of Retinal Ganglion Cells

**DOI:** 10.3390/ijms21020451

**Published:** 2020-01-10

**Authors:** Kim Tuyen Nguyen-Ba-Charvet, Alexandra Rebsam

**Affiliations:** Institut de la Vision, Sorbonne Université, INSERM, CNRS, 17 rue Moreau, F-75012 Paris, France

**Keywords:** retinogenesis, retinal progenitor cell, fate control, competence, stochastic, development, RGC subtype, albinism

## Abstract

Across all species, retinal ganglion cells (RGCs) are the first retinal neurons generated during development, followed by the other retinal cell types. How are retinal progenitor cells (RPCs) able to produce these cell types in a specific and timely order? Here, we will review the different models of retinal neurogenesis proposed over the last decades as well as the extrinsic and intrinsic factors controlling it. We will then focus on the molecular mechanisms, especially the cascade of transcription factors that regulate, more specifically, RGC fate. We will also comment on the recent discovery that the ciliary marginal zone is a new stem cell niche in mice contributing to retinal neurogenesis, especially to the generation of ipsilateral RGCs. Furthermore, RGCs are composed of many different subtypes that are anatomically, physiologically, functionally, and molecularly defined. We will summarize the different classifications of RGC subtypes and will recapitulate the specification of some of them and describe how a genetic disease such as albinism affects neurogenesis, resulting in profound visual deficits.

## 1. Introduction

**Figure 1:**
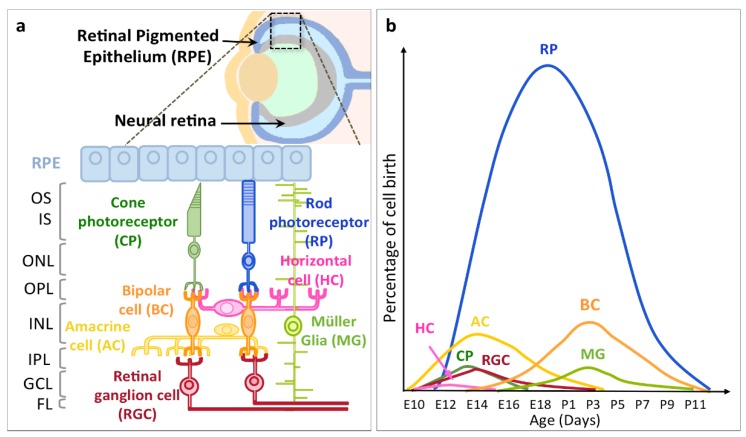
Layer distribution and genesis of the different retinal cell types. (**a**) Schematic representation of the eye with an enlargement on the neural retina and the retinal pigmented epithelium (RPE) showing all the retinal cell types and their organization into layers. Inner segments (IS) and outer segments (OS) of photoreceptors. The outer nuclear layer (ONL) contains soma of cone photoreceptors (CP) and rod photoreceptors (RP). OPL: outer plexiform layer. The inner nuclear layer (INL) contains soma of Müller glia (MG) and different interneurons: amacrine cells (AC), horizontal cells (HC) and bipolar cells (BC). IPL: inner plexiform layer. The ganglion cell layer (GCL) contains retinal ganglion cells (RGC). The fiber layer (FL) contains RGC axons. Adapted from Cepko et al., 2014 [19]. (**b**) Time course of differentiation of the retinal cells generated during mouse development illustrating the early differentiation of postmitotic RGCs. E: embryonic days, P: postnatal days. Adapted from Young et al., 1985 [12].

Retinal ganglion cells (RGCs) are the sole output neurons from the retina and thus integrate and transmit all visual information to the brain. How are these RGCs generated during development? We will first review the main sequences of retinal neurogenesis, and how multipotent retinal progenitor cells (RPCs) can generate the seven retinal cell types. These cells populate different layers. From the apical side of the eye, the outer nuclear layer (ONL) is composed of cone and rod photoreceptors. Then, the inner nuclear layer (INL) comprises the interneurons (bipolar, horizontal and amacrine cells, and Müller glial cells (MG)). Finally, The ganglion cell layer (GCL) contains RGCs. (Figure 1a). The histogenesis of the neural retina proceeds with RPCs within the germinal zone located at the apical surface near the retinal pigmented epithelium (RPE), giving rise progressively to all seven retinal cell types in appropriate proportions and a specific order corresponding to the layer order. We will discuss how extrinsic and intrinsic factors control this neurogenesis and their relative contribution. Furthermore, we will examine more in detail how the specific combination of transcription factors will determine the fate of RGCs. Strikingly, despite generic features, RGCs also acquire subtype-specific properties that are strictly related to particular functions. So far, in mice, 30 to 40 subtypes of RGCs have been characterized based on various criteria [1,2] that we will review here. While these RGC subtypes are at the basis of the multiple visual functions, the mechanisms underlying this diversity are still unclear, but we will discuss the few studies addressing the differentiation of selected RGC subtypes.

## 2. Timing of RGC Neurogenesis

All vertebrate retinal neurons are produced in a definite temporal order from a pool of multipotent progenitor cells (Figure 1b) [3,4,5,6,7]. The timing of retinal neurogenesis is an integral part of the differentiation. Rat RPCs, when isolated at E14, differentiate into RGCs, but form rods when isolated at P1 [8]. Later, Brzezinski et al. showed that RPCs could be divided into at least two categories: early RPCs that are Ngn2+ and give rise to RGCs, and late RPCs that are Ascl1+ and differentiate into the other types of retinal neurons [9]. In 1985, Young adapted birth dating data from earlier studies [10,11] to his results from tritiated-thymidine injection and produced the first differentiation time-course of mouse retinal neurons (Figure 1b) [12]. It showed that RGCs start to differentiate after E10 and that they represent 2.7% of the retinal cells, the most numerous one being rods (72.3%). In vertebrates, RGCs are always the first-born retinal neurons or amid the earliest. Moreover, they were often around the future localization of the optic nerve. For instance, in chick, the first RGCs were found around the optic nerve head at E2 [13], while in the zebrafish embryo, the first BrdU-positive postmitotic cells were detected at 28 h post-fertilization in the GCL of the ventronasal retina, next to the optic stalk [14]. In *Xenopus laevis*, similar tritiated-thymidine injection studies have situated the birthdate of RGCs between stages 24 and 29 [4,15]. In mice, cumulative labeling with BrdU injection and rhodamine-dextran tracing showed that the first RGCs appear between E11 and E12 [16]. Both in chick and mice, first-born RGCs initially form a small patch around the future optic nerve head in the central retina [13,16]. Thus, RGC differentiation starts in the central retina and then proceeds towards the periphery of the retina. Finally, in the rhesus monkey, RGC genesis begins at E30 and continue until E70 [17]. In the human and mouse early embryonic retina, transcriptomic results show exceptional analogy in the developmental stages, and human RGC neurogenesis starts at D52 [18].

## 3. Extracellular Differentiation Signals

What are the triggers for the RPCs to switch from symmetric amplifying division to asymmetric division and differentiate into RGCs? After the initiation of neurogenesis, RPCs are thus confronted with two choices: to produce a post-mitotic neuron or to remain in cycle as a progenitor cell. The coordination of cell cycle exit and re-entry is essential, in order to ensure a balanced production of early versus late retinal cell types, but how are these events regulated?

Various studies in different species have pointed toward extracellular differentiation factors. For instance, in vitro, isolated early RPCs can develop into RGCs [8,20] or cones [21]. However, when a few early RPCs are mixed with postnatal RPCs in vitro, the young progenitors differentiate into rods instead of RGCs [22]. Moreover, signals from surrounding tissue control the spread of neuronal differentiation across the zebrafish retina [23].

Extrinsic factors can regulate RGC differentiation at two levels. They can provide feedback inhibition to control RPC cell fate. For instance, in vitro, rat amacrine cells influence RPC differentiation [24]. Besides, the presence of feedback signals controlling RGC number has also been shown in the frog [25]. Still, in vitro, E9 chick retinal cells inhibit RGCs differentiation of E4 retinal cells. This effect can be reversed by depleting the E9 retinal cells [26]. Ablating RGCs in vivo in the GCL at E4 in chick leads to an anachronic production of RGCs, rescued by exogenous nerve growth factor (NGF) [27]. Thus, it has been postulated that NGF, expressed in RGCs, was regulating the production of RGCs in chick, by signaling through the p75 receptor present on the young migrating RGCs [27,28]. Nevertheless, contrary to the chick, in mice, migrating RGCs expressed TrkA, while p75 is present in the GCL [29], advocating that the regulation of RGC number is different in birds and mammals. In vivo, Atoh7:GFP RPCs transplanted in the *Lakritz* zebrafish mutant (*Atoh7*^−/−^) generate two RGCs instead of one when transplanted in a wild-type zebrafish, suggesting that the environment can somehow affect the regulation of RPC fate [30]. Hence, extrinsic factors must play a role in neurogenesis and RGC differentiation, and we will review the different extracellular molecules identified. 

### 3.1. Notch

Notch is a multifunctional signaling pathway involved in numerous physiological and pathological processes during development and in adulthood [31]. In the developing retina, Notch signaling maintains neural progenitor cells by a lateral inhibition signal that eases progenitor cell growth and inhibits neuronal differentiation [32,33]. Two types of ligands have been described that bind to the Notch receptor, *Delta-Deltalike*, and *Serrate-Jagged-Lag1* [34]. Ligand-receptor binding activates a sequence of proteolytic actions, that liberates the receptor intracellular domain to then build a multi-protein complex with Maml and Rbpj, which translocates to the nucleus and regulates gene expression [35]. Throughout retinal development, this Notch protein complex directly controls Hes1 and Hes5, two anti-proneural basic helix-loop-helix (bHLH) transcription factors (TFs) that block neurogenesis [36]. Three *Delta-like* genes are expressed in the retina, *Dll1*, *Dll3* and *Dll4* [37], but studies using conditional knock-out mice revealed that only *Dll1* and *Dll4* participate in retinal development [38,39]. Most importantly, loss of function for Notch pathway components, including Notch1 [40,41], Rbpj [42,43], Delta-like1 [38] and Hes1 [44], as well as pharmacological inhibition of Notch signaling leads to early cell cycle exit, amplified retinal neurogenesis, and a particular excess of RGCs. Conversely, misexpression of Hes1 or Notch blocks RPC differentiation [45,46,47,48]. Recently, Ha et al. suggested that part of the Notch signal comes from two different sources. Notch signaling from the RPE induces RPC proliferation, while the one from the GCL inhibits RGC differentiation [49]. Surprisingly, although the role of Notch signaling in the retina has been studied for more than two decades, the expression patterns of the different components of Notch signaling in the different retinal cells and their changes during development are still confusing [49,50,51]. Last but not least, it is noteworthy to mention that epigenetic mechanisms regulate retinal Notch signaling. In the zebrafish retina, the histone deacetylase and the Tets enzymes control the Notch pathway [52,53]. Moreover, Brm, an enzyme responsible for chromatin remodeling, has been shown to block Notch signaling [54]. 

### 3.2. Sonic Hedgehog

The hedgehog (Hh) family of morphogens encodes secreted proteins essential for cell fate decisions during embryogenesis and to maintain tissue homeostasis in most species. The first member of this family, Hh, was identified in drosophila [55], followed by its vertebrate orthologs Sonic Hh (Shh), Indian Hh and Desert Hh [56]. Hh proteins bind to Patched (Ptc) [57], which will trigger Smoothened (Smo) therefore inducing signal transduction [58,59]. The Hh signaling pathway is one of the main regulators of retinal development. It has been implicated in many steps from optic disk development [60] to proliferation [61], laminar organization [62] and RGC axon guidance [63]. Shh was found in RGCs in mice [61], zebrafish [64], frog [65] and chick [66]. Hh signaling from newly generated RGCs is one of the signals inducing RPC proliferation. When Shh is removed from RGCs, retinas are much smaller [60,61,62,67]. In mouse as well as in chick retina, the Shh pathway acts as a negative feedback controller of RGC neurogenesis. More precisely, Shh from young born RGCs regulates RGC differentiation within a normal period of retinogenesis [66,68].

Interestingly, the *sonic-you (syu)* mutant zebrafish, which has a deletion in the *Shh* gene, exhibit delayed photoreceptor and RGC differentiation [69,70]. Another study where Hh signaling was blocked showed that both cell cycle exit and RGC maturation were inhibited. The difference between mice and zebrafish may originate from the different sources of Shh. In mice, Shh is only secreted by RGCs, while in the zebrafish, Shh is also detected in the RPE [69]. An alternative explanation could be that the photoreceptor delay is secondary to the RGC differentiation defect [71].

### 3.3. Fibroblast Growth Factors

Much less is known about the role of fibroblast growth factor (FGF) in retinal neurogenesis. Nevertheless, several studies in different species showed that FGF signaling during retinogenesis contributes to RPC fate decisions. The first evidence came from the chick retina in vitro. When using a protein kinase inhibitor to block FGF signaling, RGC neurogenesis was delayed. Conversely, FGF1 but not FGF8 treatment stimulates RGC differentiation [72]. Overexpression of FGF2 in Xenopus RPCs led to a 35% increase in the number of RGCs [73]. In the chick and the zebrafish retina, different FGFs were involved. FGF8 activated retinal neurogenesis from the optic stalk. Moreover, both FGF3 and FGF8 regulate the secretion of Shh from RGCs [74]. It is then possible that these growth factors stimulate RPC cell cycle exit and differentiation into RGCs through Shh signaling [64,74,75]. 

In conclusion, these studies exploring the role of extrinsic factors in RGC neurogenesis clearly show that these molecules are essential to regulate retinal cell differentiation. Nevertheless, the mechanism at stake is likely different between fish, birds and mammals.

## 4. Competence and Stochastic Model of Retinal Cell Fate

Three decades ago, retinal lineage analysis in several species, revealed that early RPCs were able to give rise to clones containing highly variable cell types and were thus multipotent [3,4,5,6]. However, clones at early time points are usually larger than later ones, [3,4] and late RPCs in rodents mainly generated a restricted number of retinal cell types [3]. For instance, very few RGCs are produced after P0 [3,6]. At that period, the “competence model” [76] suggested that early RPCs were able to give rise to all retinal cell types, then over time, RPCs were going through a deterministic cascade of competence state, changing the ability of RPCs to produce certain retinal cell types as in drosophila neuroepithelial cells [77]. 

What could trigger this temporal control of cell fate? One possibility is that extrinsic signals from the environment could modulate this competence. However, graft experiments showed that early RPCs maintain their multipotency even in older environments [24,78,79]. Furthermore, in vitro explants of RPCs taken at different time points recapitulate the size and composition of temporally similar clones in vivo [80]. Thus, it is more likely that intrinsic mechanisms regulate the competence of RPCs and the future fate of their progeny. Interestingly, when the progeny of RPC clones was studied in detail both in vitro [80,81] and in vivo using time-lapse imaging [82], it revealed a large variety of clones in size and fate composition even at a given developmental time, illustrating a substantial variability in RPC division and competence. It became less evident to link a clear competence state of RPCs with a specific developmental time. 

One possibility derived from mathematical models is that the proliferation and fate of RPCs could be partially stochastic [80]. In this model, individual RPCs are multipotent, but the probability that they produce an early retinal cell type (such as RGC) decreases over time, whereas the probability of producing late retinal cell types (such as rod photoreceptor) increases over time. Furthermore, their type of division could also be probabilistic. Initially, there is a high probability for proliferative division (to divide symmetrically, generating two progenitors), then, a higher probability for asymmetric division (generating one progenitor and one differentiated cell). Later on, differentiative divisions of RPCs will likely give rise to two differentiated cells (similar or different). In this model, there is still a slight probability of generating two differentiated cells at early stages and two progenitors at late stages, accounting for lineage analysis experimental data observed [80,82]. Furthermore, the general order of cell birth is not conserved within individual lineages. In some cases, for example, a bipolar cell was generated before an amacrine cell within the same clone. Also, Müller glia can be produced after rod photoreceptors in a few clones, suggesting that RPCs do not lose their neurogenic potential once they have generated a glial cell [80]. However, the fate determination of retinal cells is probably not purely stochastic as the frequency of some clonal composition is much higher than what would be expected (such as some same type pairs, or the fact that the sister cell of a RGC is often a RPC), suggesting that these stochastic events can be skewed in certain directions. This stochastic model, with changes in probabilities over time, considers the majority of cells produced but also experimentally observed oddities. 

## 5. Factors Controlling the Competence of Progenitors

**Figure 2:**
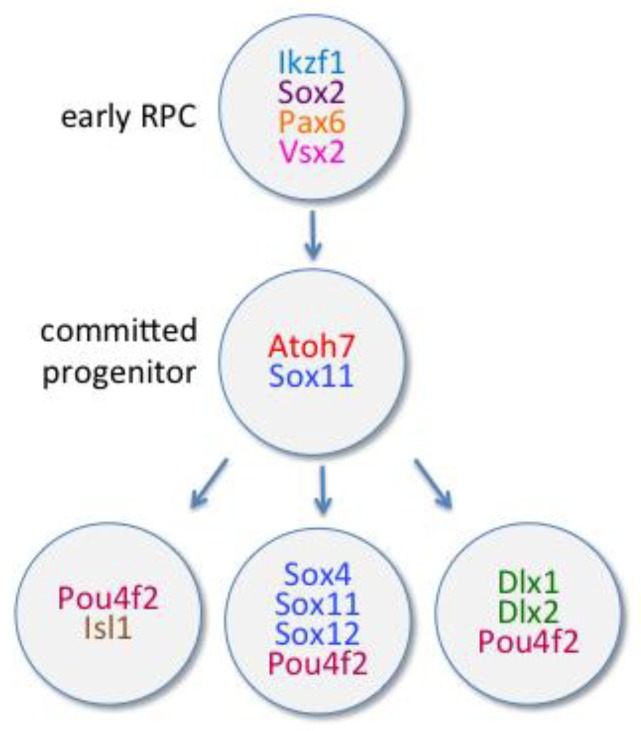
Transcription factors participating in the specification of RGCs from early retinal progenitor cells (RPC) to committed progenitors that will later produce postmitotic RGCs.

A perfect molecular candidate to control the changing states of competence or probability needs to be expressed in RPCs and to change over time. One such factor is Ikaros family zinc finger protein 1 (Ikzf1 or Ikaros), a vertebrate ortholog of hunchback in drosophila. Ikzf1 is expressed in early proliferative RPCs [83] (Figure 2), and its lineage was determined using ikzf1-cre mouse lines can label all retinal cell types (early and late) in an unbiased manner [84]. However, when ikzf1 is overexpressed, RPC production is biased toward early fates, and in *ikzk1* mutant mice, fewer early-born cell types are produced [83]. Another factor, Casz1, is a vertebrate ortholog of drosophila temporal identity factor castor. Casz1 is expressed in mid/late stage RPCs in murine retina and controls the fate of these mid/late neurons [85]. Indeed, the conditional deletion of Casz1 in RPCs increases the production of early-born neurons at the expense of later-born ones [85]. However, no change in clonal size distribution was observed in loss or gain of function experiments suggesting that Casz1 alters RPC fate independently of proliferation or cell death [85].

Furthermore, the ectopic expression of Casz1 promotes the production of mid/late-born retinal neurons. Interestingly, casz1 is repressed by Ikzf1, similarly as in drosophila with castor and hunchback [85]. Thus, the same molecules are controlling temporal fate patterning in mouse retina and drosophila neuroblasts. However, only two homologs of the drosophila temporal fate transcription cascade have been characterized as temporal fate transcription factors (TFs) in mice. It remains to be seen whether other orthologs such as Krüppel or Pdm that are intermediate in the TF cascade in drosophila between Hunchback and Castor, are also part of the same gene regulatory network in mice retinogenesis [77]. One difference, though, is that in drosophila, the temporal fate patterning is completely deterministic while it is probabilistic in the mouse. Hence, other molecular players are likely at stake.

## 6. Intrinsic Control of RGC Specification

### 6.1. Transcription Factors

These competence-controlling factors could change the probability to express (or not) particular sets of genes that control the specification of the different retinal cell types. In this review, we will focus on the TFs that control the generation of retinal ganglion cells. Other sets of TFs are also controlling the generation of the other retinal cell types and are reviewed in [19,86,87]. All RPCs express during the early proliferative phase of retinal development, the TFs Pax6, Sox2 and Vsx2 (previously named Chx10) (Figure 2), that are important for their multipotent state and their self-renewal [88,89]. At this time Vsx2 inhibit the basic helix–loop–helix (bHLH) TF Atoh7 (formerly Ath5 or Math5 in mice) and Vsx1 [90]. Then, Vsx2 is downregulated in almost all RPCs and Atoh7 starts to be upregulated in RPCs that will become RGCs (Figure 2), as shown using lineage tracing in the zebrafish retina [90].

#### 6.1.1. Atoh7

Atoh7 is transiently expressed in the mouse retina starting at E11 [91] and is necessary for the generation of RGCs (Figure 2), but not sufficient on its own [92,93,94]. Loss of Atoh7 leads to an 80% reduction in RGCs in the mouse [92] and an increase in amacrine cells and cone photoreceptors [92,93]. In the zebrafish Atoh7 mutant (*lakritz*), there is an almost complete loss of RGCs [95]. Lineage analysis of Atoh7 expressing cells shows that Atoh7 cells give rise to multiple retinal cell types, including RGCs, amacrine, horizontal, and photoreceptor cells in mouse and zebrafish [30,96,97,98]. In the absence of Atoh7, early RPCs fail to exit the cell cycle and keep proliferating [94,97]. The expression of Atoh7 is spatiotemporally regulated by the competition or cooperation of different bHLH proteins such as Ngn2, Hes1, NeuroM and Atoh7 itself on evolutionarily conserved sequences of Atoh7 promoter [99].

#### 6.1.2. POU Domain, Class 4, Transcription Factors

Atoh7 is the main TF regulating the fate of RGCs, but several other TFs, downstream of Atoh7 are also crucial for the specification of RGCs. For instance, Atoh7 functions upstream of the family of POU domain, class 4, transcription factors (Pou4f, previously named Brn-3) to promote retinal ganglion cell development (Figure 2) [100]. During mouse retinogenesis, Atoh7 is responsible for the initiation of Pou4f2 (previously Brn-3b) expression in postmitotic RGCs, which in turn activates the expression of Pou4f1 (Brn-3a) and Pou4f3 (Brn-3c) in respectively around 80% and 20% of developing RGCs [100,101,102,103]. The expression of Pou4f2 in differentiated RGCs is then maintained by a combination of auto-activation and feedback regulation by Pou4f1 and Pou4f3 [100]. Pou4f2 is necessary for the terminal differentiation and survival of most RGCs but was not thought to be required for their initial specification [102,104,105,106]. However, later experiments using *Pou4f2b*^−/−^ mice showed that these cells produced amacrine and horizontal cells as well as late-born RGCs, but few early-born RGCs. Thus, Pou4f2 can act as a RGC specifier gene by promoting RGC differentiation and by suppressing non-RGC differentiation programs (Figure 2) [107]. *Pou4f3*^−/−^ mice show no visible retinal defects [104,108], but Pou4f3 is required for RGC differentiation and can partially compensate for the loss of Pou4f2 as Pou4f2/Pou4f3 double knockout mice were more severely affected than Pou4f2 knockout mice [109]. Loss of Pou4f1 leads to a very modest RGC loss and an increase in the ratio of bistratified to monostratified RGCs, suggesting a role for Pou4f1 in the RGC subtype specification, rather than an early role on RGC specification [110]. Pou4f2 also regulates directly Shh [106] and Tbr2 (also called eomes or eomesodermin) [111].

Besides, Atoh7 regulates the expression of Insulin gene enhancer protein 1 (Isl1 or islet1), a LIM homeodomain TF that defines a distinct but overlapping subpopulation of RGCs with Pou4f2 [112,113,114,115]. Similarly, Pou4f2 and Isl1 cooperate for RGC differentiation and survival [102,104,105,108,113,116] and function downstream of Atoh7 for RGC specification. Indeed, the ectopic expression of Pou4f2 and Isl1 in the Atoh7-null retina is sufficient to rescue RGC specification [117]. Thus, the Atoh7-Pou4f2/Isl1 pathway specifies a population of RGCs (Figure 2). In the human embryonic retina, ATOH7 and POU4F2 are highly expressed between D52 and D57 [18], suggesting potentially conserved mechanisms.

#### 6.1.3. Dlx Family of Transcription Factors

The distal-less homeobox family of TFs is also important for RGC differentiation and survival, especially Dlx1 and Dlx2. A Dlx regulatory region is a direct target of Atoh7 in chick retina [118]. Indeed, *dlx1*/*dlx2*^−/−^ mice have reduced RGCs due to the enhanced apoptosis of late-born RGCs [119]. Dlx1 and Dlx2 were also identified as transcriptional activators of *Pou4f2* expression (Figure 2) supported by in utero retinal electroporation of *Dlx2* and siRNA-mediated knockdown of Dlx2 in primary embryonic retinal cultures [119]. Furthermore, *dlx1*/*dlx2*/*pou4f2* triple knockout mice show defects that are stronger than the combined effect of *pou4f2*-KO and *dlx1*/*dlx2*^−/−^, with an almost complete loss of RGCs and a marked increase in amacrine cells in the ganglion cell layer (GCL) [120]. Therefore, the regulation of Pou4f2 by Dlx1 and Dlx2 is required for RGC differentiation in the vertebrate retina [120]. 

#### 6.1.4. SoxC Family of Transcription Factors

Lastly, another set of TFs has been implicated in RGC specification, the SoxC family of proteins. Early progenitor cells express Sox11. Loss of Sox11 resulted in the delayed initiation of ganglion and cone cell neurogenesis without affecting proliferation. However, postnatal development was normal, only a moderate reduction of RGCs was observed, possibly due to the redundant activity of Sox4, which expression starts at postnatal ages [121,122]. However, loss of both Sox4 and Sox11 in the retina resulted in the absence of RGCs without affecting Atoh7 or Pou4f2 (Figure 2). As Atoh7 removal abolished the expression of Sox4 and Sox11 and overexpression of Sox4 and Sox11 stimulated Pou4f2 expression in vitro, Sox4 and Sox11 function downstream of Atoh7 while upstream of Pou4f2 to regulate the development of RGCs [122].

#### 6.1.5. Other Regulators

Recently, many studies have used bulk RNA sequencing [18,123] or single-cell RNA sequencing to study the development of the retina in both mice [124,125] and humans [126]. Some potential new regulators have emerged, such as Eya2, a protein phosphatase involved in protein-protein interactions and post-translational regulation that is a downstream target of Atoh7 and can control Pou4f2 expression in vitro [123]. Also, TFAP2D is a potential candidate found in the human retina, as it is a transcription factor expressed in RPCs and developing RGCs in the human retina from 5 gestational weeks [126], and is also expressed in mouse retina from E13.5 to E16.5 [127]. A role for these molecules in determining RGC identity remains to be shown by functional validation.

In summary, three parallel but potentially cross-regulatory transcriptional pathways seem to play a role in RGC differentiation involving either Atoh7-Pou4f2/Isl1, Atoh7-Dlx1/Dlx2-Pou4f2, and Atoh7-Sox4/Sox11-Pou4f2 (Figure 2). Transcriptomics is revealing new transcription factors expressed in RPCs that could potentially direct RGC fate (or the fate of other retinal cell types). However, the functional validation of most of these genes is still required yet impossible in humans. The use of retinal cells generated from human induced pluripotent stem cells (iPSCs) could address this problem, as they are amenable to genetic manipulation [128].

### 6.2. MicroRNA

MicroRNAs (miRNAs), small nucleotide RNAs that influence gene expression through post-transcriptional regulation of mRNA translation and degradation, have recently emerged as essential regulators in RGC neurogenesis [129,130]. 

Loss of function experiments in Xenopus embryo suggested that miRNAs could regulate the timing of retinal neurogenesis [131]. Different conditional knock-out mice were used to study Dicer function in the retina and allowed to conclude that miRNAs, and in particular let-7, miR-125, and miR-9, regulate the transition between early RPCs and late RPCs [132,133,134,135,136]. Indeed, early RPCs in Dicer mutant mice did not express the TF Ascl1 and therefore did not switch to the late progenitor state, which resulted in an overproduction of RGCs [132]. Moreover, in vitro re-aggregation experiments showed that this miRNA function is cell-autonomous [135]. In the next RGC differentiation step, Pou4f2 (Brn-3b) is necessary for the terminal differentiation and survival of most RGCs [120]. This TF seems to be down regulated by miR-23a and miR-374 [137].

The exact role of miRNAs has been difficult to decipher, partly because of their instability [138]. Moreover, there are many candidate target genes for a single miRNA [139]. Finally, as miRNA are fine regulators, loss of function studies often showed slight phenotypes [140]. In order to overcome these problems, many studies have chosen to analyze the loss of miRNA regulation. For instance, in the retina, many results came from disrupting the pre-miRNA processing enzyme Dicer.

Thus, a *Dicer* conditional-knockout (cKO) suppressing miRNA in RPCs showed pathfinding defects in the optic chiasm [141]. Based on this phenotype, the authors hypothesized that Dicer could regulate axon guidance at the midline. However, these *Dicer* cKO also presented severe microphtalmia even though the eye structure remained normal [141]. Therefore, the pathfinding default might also, in part, results from defects in RGC neurogenesis.

Here we have reviewed the different regulators of RGC neurogenesis extensively. Nevertheless, even if we have mentioned epigenetic regulation of the Notch pathway, we should not forget that variations in histone modifications, changes in DNA methylation and hydroxymethylation patterns have been shown to regulate neurogenesis (reviewed in [142]). For instance, in addition to inhibiting Notch signaling, Brm, an enzyme responsible for chromatin remodeling, also induces cell cycle exit and enables Pou4f2 expression in vitro [54].

## 7. The Ciliary Marginal Zone, a Source of Neurogenesis in Mammals

**Figure 3:**
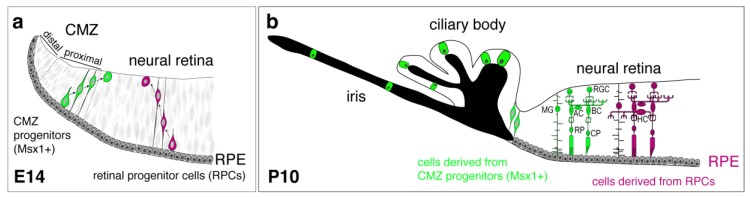
Schematic representation of lineage tracing from progenitors of the ciliary marginal zone (CMZ) or neural retina in mouse. (**a**) At E14, retinal progenitor cells (RPCs) (magenta) are the main contributor to retinal neurogenesis, but Msx1+ progenitors (green) in the proximal CMZ migrate towards the neural retina and contribute to retinal neurogenesis at the periphery of the retina. (**b**) At P10, cells derived from Msx1+ progenitors (green) produce most retinal cell types, although with a different ratio than cells derived from RPCs (magenta). Also, Msx1+ progenitors (green) contribute to non-pigmented epithelial cells of the ciliary body and the cells in the iris. RPE: retinal pigmented epithelium, RGC: retinal ganglion cells, BC: bipolar cells, AC: amacrine cells, MG: Müller glia, RP: rod photoreceptors, CP: cone photoreceptors, HC: horizontal cells. Adapted from Marcucci et al., 2016 [151] and Bélanger et al., 2017 [152].

Now that we described the neurogenesis in RPCs, we will discuss another source of neurogenesis that originates in the ciliary marginal zone (CMZ), the transition area between the neural retina and the RPE (Figure 3a). The progenitor cells in the CMZ contribute to the establishment of the neural retina by producing new neurons during development and throughout life in many lower vertebrates [5,143,144,145,146]. Many studies in non-mammalian vertebrates have pointed out a role of Müller glia and the CMZ as a source of stem cells for adult neurogenesis in the retina, particularly upon lesion [146]. Thus, the addition of neurons generated from the CMZ participates in eye growth [147] in addition to the neurons generated from the main RPCs that are differentiating in a central to peripheral wave in the neural retina [76,145,148,149].

However, in rodents, the CMZ was not considered a stem cell niche for the neural retina. Indeed, previous experiments had shown that the Pax6 removal in the distal CMZ, using a *Pax6^flox/flox^*; *Tyrp2-Cre* cKO did not impair the neuronal differentiation of the retina [150]. However, recent evidence has shed light on the CMZ contribution to retinogenesis. Time-lapse imaging using a Zic2-GFP reporter mouse line labeling a subset of proximal CMZ progenitors showed that some CMZ cells were migrating to the peripheral neural retina to become RGCs (Figure 3a). Furthermore, Cyclin-D2 was necessary for the generation of some of these RGCs, especially for the ipsilateral Zic2-positive RGCs [151]. The definitive contribution of the CMZ to the different retinal cell types was determined using a lineage tracing approach with Msx1, a TF selectively expressed in the proximal area of the CMZ [152]. Most cells derived from Msx1 progenitor cells were located in the non-pigmented epithelium of the ciliary body and in the iris (Figure 3b).

Nevertheless, some Msx1 progenitor cells can differentiate during embryogenesis into the different types of retinal cells (RGCs, photoreceptors, Müller, amacrine and bipolar cells) (Figure 3b). However, the proportion of retinal cells produced by CMZ-derived Msx1 progenitors differs from the retinal cells produced by the main neural RPCs, with fewer photoreceptors produced for instance [6]. Also, Msx1 progenitors seem to generate selectively part of the ipsilateral RGCs [151], suggesting a different bias in the production of retinal cells from CMZ progenitors compared to neural RPCs. Furthermore, CMZ-derived retinal cells are located at the periphery of the retina and not the central retina [152] (Figure 3b), suggesting that centrally-located retinal cells are generated from RPCs and that retinal progenitors or cells do not migrate over significant distance.

In the future, it would be interesting to identify the mechanisms of cell fate specification for CMZ progenitors, and whether they differ from the main RPCs. Overall, the proximal region of the CMZ in mice is also able to contribute to the generation of the neural retina during development (Figure 3). However, murine CMZ cells are unable to generate neural retinal cells postnatally and in the adult [152].

## 8. RGC Migration

**Figure 4:**
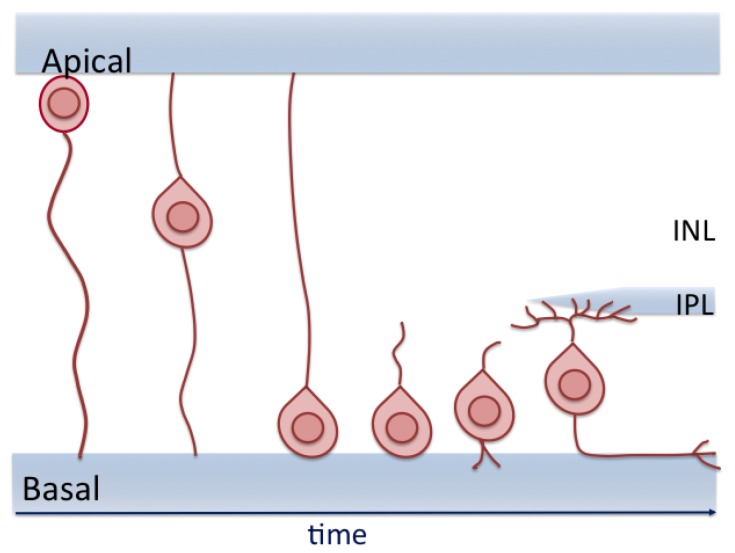
The newborn retinal ganglion cell (RGC) translocates from the apical part of the retina toward the future ganglion cell layer at the basal side. During the entire process, the cell is attached to both sides of the retina. After the migration, the RGC adjusts its position while losing its apical process and send its axon to the optic nerve. INL: inner nuclear layer, IPL: inner plexiform layer. Adapted from Amini et al. [159].

After exiting the cell cycle at the apical side, postmitotic RGCs, migrate toward the GCL all along the apico-basal axis where they will develop their axon (Figure 4). Several decades ago, the migration pattern of RGCs was already described in rodents using Golgi staining and electron microscopy [153,154]. These studies hypothesized that RGCs migrated by translocating their soma. In the chick, the R4 antibody labeling RGCs described a transient bipolar RGC spanning the entire thickness of the retina around E4. In older embryos, these bipolar RGCs disappear and only unipolar RGCs in the GCL were immuno-stained [155]. Later, time-lapse video-microscopy studies on the zebrafish retina confirmed that the RGC soma was translocated to the GCL while they remained attached to the apical and basal surface of the retina, hence presenting a bipolar shape (Figure 4) [30,156,157].

Little is known about the molecules regulating RGC migration. Recently, it has been shown in mice that the β1-Integrin laminin receptor with Cas signaling-adaptor proteins are necessary for RGCs to find their way to the GCL and arrange into a single cell layer [158].

Thus, this migration of young postmitotic RGCs is required for their final maturation and for the proper organization of retinal cells into layers.

## 9. Classification of RGC Subtypes

In the next section, we will discuss how RGCs have been classified by anatomy, function and molecular signature and also how their pathfinding and targeting also discriminate the different subtypes.

With rare exceptions, all RGCs have in common their cell bodies positioned in the GCL (Figure 1a). They also develop their dendrites in the INL and their axon toward the brain. Finally, vertebrate RGCs can be identified by pan-RGC markers such as the cell surface protein Thy1 [160], the RNA-binding protein RBPMS [161] and the TFs of the Pou4F family [103,110].

### 9.1. Morphological Criteria

Santiago Ramon y Cajal was a pioneer at describing the different neurons in order to understand neuronal circuits, and characterized different types of RGCs in detail [162]. Nevertheless, the classification has tremendously changed over the past few years as a result of the advances in new technologies such as single-cell transcriptomics, functional imaging, or large-scale electron microscopy [2,163,164]. Here our purpose is not to debate what defines an RGC cell type. We will recapitulate studies that have described the diversity of RGCs according to different criteria in order to highlight how much is left to decipher the mechanisms of RGC differentiation.

Even if, for a long time, the criteria of morphology and function have been used to look for the different types of RGCs, anatomical classifications have led the field due to the technical limits of defining functional subtypes with receptive field studies. The categorization of RGC dendritic morphologies in the mouse retina based on combined microinjection of Lucifer Yellow and DiI revealed 11 to 14 types of RGCs [165,166]. This classification was not very different from the one established with a genetic method using alkaline phosphatase as a histochemical reporter that yielded 12 different types of RGC [167]. Later, the injection of neurobiotin increased the number to 22 [168]. In the last decade, the addition of computational techniques enhanced spatial precision and, combined with the quantification of the dendritic arbor, categorized 15 types of RGCs [169].

### 9.2. Functional Criteria

However, based on their function, 21 types of RGCs were found (reviewed in [1]): the ON-OFF direction-selective ganglion cells (ON-OFF DS RGC) (4 types) [170], the ON direction-selective ganglion cells (3 types) [171], the alpha RGCs that are Smi-32+ in mice (3 types) [172], the intrinsically photosensitive RGCs (ipRGCs) that expressed melanopsin (5 types). These neurons have the particularity to participate in the synchronization of the circadian oscillator [173]. In mice, 13% of the RGCs close to the center of the visual field are Local Edge Detectors (LED) RGCs (3 types) [174,175]. These neurons correct excitation from both On and Off bipolar cells. Finally, the mouse retina also contains three types of J-RGCs named after their expression of the junctional adhesion molecule B, which selectively respond to stimuli moving in a soma-to-dendrite direction [176]. More recently, optical imaging advances have revolutionized the field (reviewed in [177]). For instance, Baden et al., found at least 32 RGC types relying on their light responses and anatomical criteria [163].

### 9.3. Molecular Criteria

**Figure 5:**
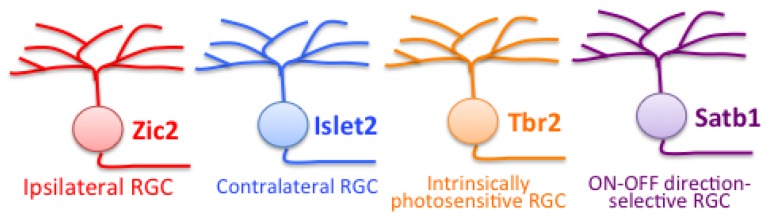
Examples of specific transcription factors that determine the identity of RGC subtypes or subclasses.

Last but not least, countless studies have classified RGCs following their molecular signature, and in particular, the presence of determining transcription factors. Recently, Sweeney et al. have proposed that almost all RGCs could be categorized in three classes depending on their expression of Isl2, Tbr2, or Satb1/Satb2 and hypothesized that these TFs are responsible for RGC functional identity [178]. Supporting this idea, Isl2 colocalizes with Smi32 a marker of alpha-RGCs [179]. Moreover, Satb1 controls the morphogenesis of ON-OFF DS RGCs, as loss of Satb1 results in mono-stratified dendrites and a lack of ON input (Figure 5) [180]. However, Satb1 does not affect the generation or survival of these ON-OFF DS RGCs [180]. Moreover, Tbr2 was found in non-overlapping RGC subtypes that project to non-image-forming brain areas such as the pretectum, suprachiasmatic nucleus, and ventral LGN, and include all melanopsin-expressing ipRGCs [181,182]. Furthermore, it is worth noting that Tbr2 is crucial for the formation and maintenance of ipRGCs (Figure 5), which exclusively express Opsin 4 (Opn4) [181], and that Pou4f2 and Tbr2 label RGCs that do not overlap [182].

The first single-cell RNA sequencing experiments have raised hope to unify the different classifications under a universal one combining molecular signature, morphology, and function [2,183]. Such a study would allow characterizing and targeting specific subtypes for modifications with unprecedented precision, as done using genetic in most invertebrates. The first study using single-cell RNA sequencing was performed on the whole retina of mice before eye-opening and categorized 40 RGC types [2]. Another study looked for new genes that could define new RGC subtypes among Parvalbumin-expressing RGCs. However, the clustering into eight subtypes did not reveal a molecule that could identify a specific RGC subtype without being expressed in other retinal cells. This work suggests that molecular signatures identifying RGC subtypes are likely composed of a combination of genes, rendering their identification and genetic modifications challenging [183].

Further studies are needed to identify a more complex molecular signature than previously expected, as many fields such as developmental biology, evolution, physiology will benefit from this unified categorization. For the moment, the categorization choice should relate to the scientific question asked. However, in the particular case of RGC neurogenesis, a robust technique combining the lineage tracing with the molecule signature is needed, as recently used in the cortex [184].

### 9.4. Brain Targeting and Pathfinding at the Optic Chiasm

#### 9.4.1. Brain Targeting

The previous classifications do not take into account the projection site of these RGC subtypes to different nuclei in the brain. In the mouse, 46 different RGC targets have been identified participating in image-forming and non-image forming visual functions [185], which is higher than the number of RGC subtypes identified so far. How can the numerous targets match the number of RGC subtypes? First, a single RGC can project to different targets. For instance, most RGCs that project to the dorsolateral geniculate nucleus, also project to the superior colliculus [186]. However, many studies following RGC targeting have relied on transgenic mouse lines expressing GFP in one specific RGC subtype or a few RGC subtypes. In most cases, these RGC subtypes project to several brain targets rather than a single target. This can result from 2 possibilities: (1) one single RGC projects to several targets that are the same for each subtype; (2) each RGC within a subtype projects to a single brain target or few targets that are not always the same. The second possibility will suggest a further subdivision of RGC subtypes depending on their targets. So far, only single axonal reconstruction is able to identify RGC morphology and clearly link it with precise targeting, but it is painstaking and usually lacks the molecular signature of these RGCs [186]. Thus, it is still an open debate (reviewed in [187,188]).

Interestingly, different RGC populations employ different strategies to achieve accurate axon-target matching. The strategy used correlates with the birthdate of RGCs and the timing of axon growth [189]. Thus, early-born and early-projecting RGCs tend to first innervate multiple targets, to then refine to the appropriate ones (usually several) while later-born and later-projecting RGCs are highly accurate in their initial targeting [189]. However, the existence of a causal relationship between RGCs birthdate and subtype identity remains to be determined. Indeed, their targeting properties could also only be related to the axonal order of arrival in their targets. In this case, early axons would have more possibilities of targeting than later-arriving ones, which would then have to compete for the remaining space.

#### 9.4.2. Pathfinding at the Optic Chiasm

Another criteria that classify RGCs into two distinct classes is their axon guidance decision at the optic chiasm: crossing or not at the midline to project to the opposite (contralateral) or same side (ipsilateral) of the brain [190,191]. This categorization into ipsilateral or contralateral RGCs comprises different subtypes regrouping RGCs that are morphologically different [186,192] and RGCs some that target different brain areas [188].

The TF Islet2 is expressed in post-mitotic contralateral RGCs (Figure 5) and represses the ipsilateral program as Islet2 knock-out mice showed an increased ipsilateral projection correlated with fewer ipsilateral RGCs [193]. Recently, the SoxC family of TF (Sox4, 11 and 12) that was previously implicated in RGC neurogenesis [121,122], was identified as a specific regulator for the differentiation of contralateral RGCs [194]. Interestingly, SoxC TFs stimulate contralateral RGC differentiation by antagonizing the Notch signaling pathway [194]. Additionally, SoxC TFs regulate molecules that are important for contralateral axon guidance such as Plexin-A1 and NrCAM [194,195].

We will focus more on the ipsilateral subclass of RGCs as their molecular characterization is currently the best described. Ipsilateral RGCs are located in the ventro-temporal retina in mice and express the TF Zic2 that defines their identity and controls the expression of the guidance receptor EphB1 and the serotonin transporter SERT [196,197]. These genes are transiently expressed, during a specific period of development [198,199,200]. While involved in the proper refinement of ipsilateral retinal axons in their brain targets, SERT is not necessary for their midline decision [197,201]. Besides, EphB1 loss of function or Zic2 knock-down in mice leads to a reduced ipsilateral projection [198,199], while ectopic expression of Zic2 or EphB1 is sufficient to induce the ipsilateral misrouting of some retinal axons [196,202]. Thus, Zic2 is essential to specify ipsilateral RGCs (Figure 5), while EphB1 controls their guidance decision at the optic chiasm. Indeed, EphB1-expressing axons are repelled at the midline by ephrin-B2, to project ipsilaterally. As previously mentioned, a RPC subpopulation within the CMZ that contribute to retinogenesis has been isolated [152]. Hence, Zic2^+^ progenitors in the CMZ seem to migrate toward the neural retina and generate ipsilateral Zic2^+^ RGCs [151]. What are the upstream regulators controlling the neurogenesis of ipsilateral RGCs? A microarray screen identified new molecular determinants of ipsilateral and contralateral RGCs at embryonic ages [203]. Among them, Cyclin-D2 (ccnd2), a regulator of the cell cycle, was upregulated in ipsilateral RGCs and also found expressed in the CMZ [151,203]. *Ccnd2*^−/−^ mice have reduced mitosis in the ventral CMZ and a diminished neurogenesis in the ventral retina affecting both the contralateral and ipsilateral RGCs [151], suggesting that Cyclin-D2 could regulate cell cycle exit and neurogenesis. Another interesting upstream candidate is Foxd1, a TF expressed in the ventral retina at embryonic stages. Foxd1 controls Zic2 expression as *foxd1*^−/−^ mice lack Zic2 expression in ventro-temporal retina whereas its ectopic expression leads to the ipsilateral misrouting of some Foxd1-expressing axons [204,205]. Furthermore, Shh regulates the specification of ipsilateral RGCs, as *Boc*^−/−^ mice presented a modification of ipsilateral and contralateral markers [206].

## 10. Altered Neurogenesis in Albinism

A genetic disease with reduced ipsilateral projection has provided some insight into the generation of ipsilateral RGCs and neurogenesis in general: albinism [190]. Albinism is a rare genetic disease characterized by hypopigmentation, affecting mainly eyes (in ocular albinism) or also skin and hair (in oculocutaneous albinism) and results in a profound visual impairment that includes very low visual acuity, photophobia, nystagmus, and abnormal binocular vision (stereopsis) [207,208,209,210]. The pigmentation defect occurring at the level of the RPE affects the retinal neurogenesis, resulting in fewer ipsilateral RGCs [211,212] and reduced numbers of photoreceptors [213] in albino mouse models, most likely at the origin of the deficits in binocular vision and visual acuity, respectively. Indeed, the delayed neurogenesis leading to the reduction in the number of ipsilateral RGCs results in a reduced ipsilateral projection and abnormal brain projection [198,211,212,214,215]. How is neurogenesis perturbed in the albino retina? Interestingly, there are fewer Ccnd2+ cells in the CMZ of albino retinas during development [151]. Thus Cyclin-D2 is an interesting candidate, upstream of Zic2 expression, and essential for the specification of ipsilateral RGCs. However, how this altered neurogenesis occurs from a pigmentation defect in the adjacent RPE is still a mystery. Several signaling pathways that could alter neurogenesis in albinism have been identified. Wnt signaling in the peripheral RPE has been implicated as a negative regulator of ipsilateral identity and Wnt2b expression is expanded in the central RPE in albino mice instead of being confined to the periphery as in pigmented mice [216]. In addition, activating Wnt signaling with lithium chloride in pigmented mice during pregnancy reduced the number of Zic2^+^ RGCs to a level comparable to albino retina. This suggests that Wnt signaling could regulate Zic2 expression [216], but the mechanisms at hand are still unknown. Furthermore, the recent discovery that the regulation of Notch signaling in the RPE impacts neurogenesis in the adjacent retina [49] opens up a new avenue of research to decipher the signaling pathways linking RPE and neural retina to regulate neurogenesis, as a means to better understand the role of extrinsic factors on neurogenesis and how it is altered in the albino eye.

## 11. Conclusions

Over recent decades, much progress has been made to improve the understanding of RGC neurogenesis and differentiation. The current view is that retinal progenitor cells in vertebrates can generate different retinal types in a stochastic manner but with a probabilistic bias for some cell types that change during development. This model could explain why all retinal cell types can be generated at any given developmental time but with a different probability, ending up with RGCs generated mostly early on and rod photoreceptors later. This stochastic model is possibly a general rule for the development of the central nervous system, as suggested by recent data on the neurogenesis of the cerebral cortex [217]. The extrinsic and intrinsic factors that regulate the cell fate determination are being isolated, with compelling evolutionary conserved factors initially identified in the more deterministic neurogenesis of the drosophila eye. However, two aspects remain to be established: (1) how the developmental expression of these factors is regulated, and (2) how the change in cell fate probabilities occur over time. The transcription factors that regulate RGC neurogenesis are identified: Atoh7 and Pou4f2 appear as the key regulators with several transcription factors in between. As RGCs are not a homogeneous population, several studies have tried to identify the molecular determinants and/or markers of RGC subtypes. This characterization was done initially by combinatorial expression of various transcription factors or markers for different types of RGCs. However, the recent emergence of single-cell RNA sequencing technology will hopefully allow the identification of new markers for RGC subtypes but also to determine their specification pathways during development. In the future, studies will undoubtedly link molecular specification of RGC subtypes with their brain connectivity to decipher the molecular mechanisms that are at hand. Finally, understanding the developmental mechanisms determining the specification of retinal cells is crucial for the efficient, targeted generation of retinal cells from induced pluripotent stem cells of patients for research on the human retinal neurogenesis and also potential therapeutic strategies. 

## Abbreviations

bHLH	basic helic-loop-helix
ccnd2	Cyclin-d2
cKO CMZ	conditional knock-out ciliary marginal zone
FGF/Fgf	fibroblast growth factor
GCL	ganglion cell layer
Hh	hedgehog
INL	inner nuclear layer
MG	Müller glial cells
NGF	nerve growth factor
ONL	outer nuclear layer
Ptc	Patched
RGC	retinal ganglion cell
RPC	retinal progenitor cell
RPE	retinal pigmented epithelium
Shh	Sonic hedgehog
Smo	Smoothened
TF	transcription factor
